# An Approach to Multi-Objective Path Planning Optimization for Underwater Gliders

**DOI:** 10.3390/s19245506

**Published:** 2019-12-13

**Authors:** Carlos Lucas, Daniel Hernández-Sosa, David Greiner, Aleš Zamuda, Rui Caldeira

**Affiliations:** 1 Oceanic Observatory of Madeira, Agência Regional para o Desenvolvimento da Investigação Tecnologia e Inovação, Ed. Madeira Tecnopolo, 9020-105 Funchal, Madeira, Portugal; rui.caldeira@oom.arditi.pt; 2 Instituto Universitario de Sistemas Inteligentes y Aplicaciones Numéricas en Ingeniería (SIANI)-Universidad de Las Palmas de Gran Canaria, Campus de Tafira, 35017 Las Palmas de Gran Canaria, Spain; daniel.hernandez@ulpgc.es (D.H.-S.); david.greiner@ulpgc.es (D.G.); 3 Faculty of Electrical Engineering and Computer Science, University of Maribor, Koroška cesta 46, 2000 Maribor, Slovenia; ales.zamuda@um.si; 4 Dom Luiz Institute, Faculty of Sciences, University of Lisbon, 1749-016 Lisboa, Portugal

**Keywords:** multi-objective optimization, underwater glider, path planning, genetic algorithm, NSGA-II

## Abstract

Underwater gliders are energy-efficient vehicles that rely on changes in buoyancy in order to convert up and down movement into forward displacement. These vehicles are conceived as multi-sensor platforms, and can be used to collect ocean data for long periods in wide range areas. This endurance is achieved at the cost of low speed, which requires extensive planning to ensure vehicle safety and mission success, particularly when dealing with strong ocean currents. As gliders are often involved on missions that pursue multiple objectives (track events, reach a target point, avoid obstacles, sample specified areas, save energy), path planning requires a way to deal with several constraints at the same time; this makes glider path planning a multi-objective (MO) optimization problem. In this work, we analyse the usage of the non-dominated sorting genetic algorithm II (NSGA-II) to tackle a MO glider path planning application on a complex environment integrating 3D and time varying ocean currents. Multiple experiments using a glider kinematic simulator coupled with NSGA-II, combining different control parameters were carried out, to find the best parameter configuration that provided suitable paths for the desired mission. Ultimately, the system described in this work was able to optimize multi-objective trajectories, providing non dominated solutions. Such a planning tool could be of great interest in real mission planning, to assist glider pilots in selecting the most convenient paths for the vehicle, taking into account ocean forecasts and particular characteristics of the deployment location.

## 1. Introduction

Underwater gliders constitute autonomous robots designed to perform multi-sensor oceanographic sampling, for long periods in large regions. They represent a valid alternative to expensive research vessels campaigns for a wide range of applications, including water quality monitoring, detection of algae bloom hazards, ocean structure characterization, pollution source identification or sea-floor mapping, to name just a few [1,2]. However, their operation nowadays still require human supervision, both for short- and long-term missions. This scheme can be suitable for simple applications, but when dealing with more complex multi-objective missions, automatic path planning tools are required; this is the main motivation of our work, contributing to the development of underwater gliders with higher autonomy and better operational capabilities for multi-objective applications.

In this work, the usage of the non-dominated sorting genetic algorithm II (NSGA-II) algorithm [3] is analysed to tackle a multi-objective (MO) glider path planning application on a complex and changing environment. The problem includes the simulation of 3D vehicle trajectories inside a dynamic depth and time varying ocean currents field.

The paper first introduces, in Section 1.1 and Section 1.2, the concepts of the main areas our work aims to contribute to, which are underwater glider path planning and multi-objective optimization; then Section 1.3 presents the literature review regarding multi-objective path planning. The proposal is presented in Section 2, including the problem definition, in Section 2.1, and methodology, in Section 2.2, applied to address the problem of glider path planning using multi-objective optimization. Section 3 describes the simulator, test scenarios, and algorithm configurations. Section 4 presents the experiments carried out and the results obtained, which are discussed in Section 5. Finally, the conclusions and some future work are outlined in Section 6.

### 1.1. Underwater Glider Path Planning

**Figure 1:**
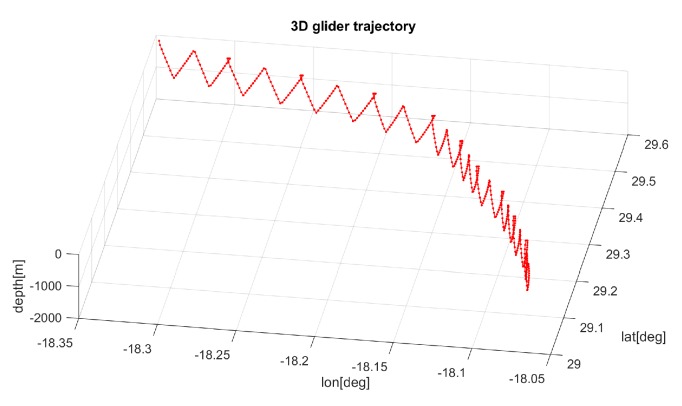
Example of a 3D generic saw–tooth glider trajectory (10 stints).

Ocean gliders are torpedo-shaped vehicles that make use of a highly energy efficient propulsion system: They modify their buoyancy and pitch angle by means of an artificial swim bladder operation combined with internal batteries displacement. The induced descend–ascend movement is transformed into effective horizontal displacement by the interaction between the water column and the vehicle hydrodynamics, resulting in a saw-tooth like trajectory profile similar to the one shown in Figure 1. Typically, the vehicle performs two or three down-up cycles, called *yo-yo’s*, between pre-defined max- and min-depths before returning to surface to start a new sequence or *stint* each 6–8 h. As energy consumption is mainly associated with the activation of the electric pump, which is responsible for the buoyancy control at inflection points, extremely long-term missions are possible with these vehicles.

During a glider mission, the control centre relies on satellite communication to upload commands to the glider when at the surface, including target waypoints or depth limits, for example. The surfacing period has to be kept as short as possible to avoid collisions with surface vessels, reduce biofouling, and decrease vehicle’s drift. After the surfacing, the glider submerges again to perform another set of stints. Although major deviations from expected trajectories occur generally at surface (due to strong currents), when the glider dives, its location can only be estimated using its on-board sensors, producing errors that grow with time. In order to reduce this uncertainty, as the ocean is a very dynamic and unknown environment, researchers have developed different strategies. Woithe et al. [4], for example, use a doppler velocity log with promising results on predicting glider position underwater; Wang et al. [5] propose a dynamic model-aided localization scheme that proved to be able to improve significantly the precision in the estimation of the vehicle position.

The main drawback of (passive) ocean gliders is that they can only reach low horizontal speeds (around 0.25 m/s). To optimize their operation, factors such as bathymetry, marine traffic or, remarkably, ocean currents, have to be anticipated, so that suitable paths are well planned in advance. As this constitutes a 4D problem (latitude, longitude, depth, and time), with variable environment conditions, the process is not so trivial.

From the traditional path planning, the strategies to achieve the best path for gliders kept evolving. An A* search procedure was done by Garau et al. [6], in areas with different length scale eddies and currents with varying intensities. However, the authors considered the usage of constant thrust power to navigate the vehicle. They also tested different start and end points across the simulation area, to find the best paths and best locations to achieve their objectives.

The usage of Lagrangian coherent structures carried out by [7] showed how those can be used to find near optimal trajectories in dynamic environments, considering a 2D glider kinematic model. They used ocean current velocities collected with an HF radar system to calculate the optimal trajectories for the vehicle.

A fast marching based approach (called FM*) was used by Petres et al. [8] in order to extract a continuous path from a discrete representation of the environment. The authors considered underwater currents, with the vehicle turning radius being added as a constraint.

The backward study of a particle tracking equation provided an optimal path in [9], with the authors stating that the system can easily handle forbidden regions (such as strong currents areas) and real obstacles that affect the ocean flow and consequently the vehicle pathway.

Further strategies to address underwater glider path planning (UGPP) under complex scenarios (static and dynamic obstacles) are discussed in [10], as the classical path planning might not be adequate. Those strategies include pointing the vehicle to the destination and make small adjustments during the mission or in adverse zones position the vehicle across the current, in order to reach a place with weaker and/or more favourable currents. The same work suggests some metrics to evaluate performance, in order to determine how well defined the path-planning arrays are, considering the mission objectives.

The work presented by [11] concluded that adequate path planning contributes to a substantial energy saving set of solutions compared to straight line trajectories, in cases when ocean currents and vehicle’s speed are comparable. The authors also point out that a straight line trajectory from start to end can only be an optimum path when the currents’ velocity does not exceed half of the vehicle’s velocity. Energy and time saved with path planned missions can be used to either increase the distance travelled and/or add more sensors.

A stochastic optimization methodology was carried out by Subramani et al. [12], focused on obtaining the best paths from the viewpoint of energy-optimal missions. They also concluded that vehicles that can change their displacement speeds can reach the programmable locations by using favourable currents, saving energy for crossing areas with unfavourable conditions.

UGPP challenge has also been tackled using evolutionary techniques, like in Zamuda et al. works [13,14], including eddy sampling applications [15].

### 1.2. Multi-Objective Optimization

In early days, due to the lack of methodologies, multi-objective optimization problems (MOOP) in general were solved by casting all objectives in one and solving it as a single objective problem [16]. Currently, MOOP are solved differently by optimizing a group of two or more fitness functions (usually in conflict [17]), without prejudice among them, until no more improvements to the current solution set could be achieved. MOOP results are (usually) a set of optimal solutions that minimize all the objective functions at the same time, or either maximize all the functions or a combination of maximization and minimization of different functions [16,18]. The set of optimal solutions is designated as Pareto front, on which a point x∈X is called Pareto optimal IFF there does not exist another point, $x^{*}\in X$, such that $F\left(x\right)\leq F\left(x^{*}\right)$, and $F_{i}\left(x\right)<F_{i}\left(x^{*}\right)$ for at least one fitness function [19]. Also, on the Pareto front, all the solutions yield the same optimal value [20].

One way to tackle MOOP and obtain solutions that converge to a Pareto front is through the usage of evolutionary algorithms (EA), very popular among researchers because of its flexibility and easy adaptation to different problems. Also, as EA are population-based algorithms, they are well suited to be used to solve MOOP in form of multi-objective evolutionary algorithms (MOEA). Currently, the usage of MOEA can be found on multiple areas [21], integrating diverse selection and estimation mechanisms.

A recent review of evolutionary multi-objective methods can be found in [22], including some that became standards when dealing with MOOP, such as the NSGA-II algorithm. The work considers three types of MOEA: Pareto-based (such as NSGA-II [23]), indicator-based (such as SMS-EMOA [24]), and decomposition-based (such as MOEA/D [25]), exposing their advantages and disadvantages. Also, choosing the right method depends on the dimension of the objective space, how many solutions should be generated, the distribution of solutions, and an a priori knowledge about the location and shape of the Pareto front.

Considering real problems of engineering, EA are currently being used for energy, electrical, structural and civil engineering, scheduling, transport, combinatorial optimization, and more, as described by Greiner et al. in [26]. However, these are just some examples, as many other problems that need to do multi-objective optimizations are using EA.

### 1.3. Multi-Objective Path Planning

One area in which multi-objective optimization is important is in path planning. Multi-objective path planning (MOPP) problems will be introduced here, including different techniques and applications discussed in the scientific literature.

MOPP is the way of finding a feasible path for a vehicle that requires travelling from a staring point A to a final point B, and accomplishes all the objectives planned for the mission. Considering the most common objective—reaching a target—other objectives for the mission might include (but are not limited to), verifying several path properties, limiting battery consumption, and crossing or avoiding specified areas [10]. Previous studies solved MOPP in different ways, for diverse types of vehicles and different environments. In [27,28], the authors used genetic algorithms (GA) to optimize both length and difficulty of the path, adding a basic path repair mechanism to make non-valid paths usable, on a 2D realm.

The genetic algorithm NSGA-II was used by Mittal and Deb [29] to perform offline path planning for an unmanned aerial vehicle, with the fitness functions being avoid collisions, no abrupt changes to the path, and not flying above a specified altitude. Also, Lee et al. [30], worked on path planning optimization for unmanned aerial vehicles, using NSGA-II combined with Nash-equilibrium to decrease the time for searching one global solution, showing that a hybridization with game theory is a valid choice to solve MOPP.

The usage of particle swarm optimization (PSO) to solve a MOPP was proposed in [31], to find smooth and shorter paths, with parameter tuning, using a 2D map. Another work using PSO was carried out by Gong et al. [32] to solve path planning on a 2D environment, incorporating previously known danger locations, and similarly in [33], where path planning in a 2D uncertain environment, with danger sources and obstacles were concurrently being considered also using PSO. A spline approach is discussed in [34], to perform MOPP with fitness functions being “path length and potential”, “obstacle hindrance”, and “visibility” of the robot. Davoodi et al. [17] described a MOPP accounting for length and clearance of the path, using path refiner operators based on geometry parameters.

Later, Bopardikar et al. [20] tried to find a path that minimizes a primary cost function subject to a bound on a secondary cost function, handling collision avoidance in all the vertexes along the path. The same work described planning in belief space, with collision probability and multi-objective, proposing one algorithm that is more accurate but with higher computation costs and another that sacrifices accuracy but is more efficient. Using the Mars Rover scenario (3D), Ref [35] developed a MOPP using A* search with real objectives (minimize difficulty, danger, elevation, and length). Usage of the Pareto front and the Pareto optimal in every step of A* was proposed by [36,37] using memetic algorithms in order to optimize both path length and smoothness.

A survey of 2016 [38], about applying nature inspired algorithms to solve MOPP, analysed the usage of particle swarm optimization, ant colony optimization, and artificial bee colony. In addition, Hidalgo et al. [39] presented a firefly-based approach to pursue MOPP considering path safety, length, and smoothness, pointing to the path planning as one of the most researched topics in robotics.

One year later, in 2017 another publication [40] reviews robot path planning techniques, with soft computing and heuristic approaches (artificial neural networks, fuzzy logic, wavelets, and genetic algorithms) replacing the classical methods (potential fields, roadmap, and cell decomposition). In same year, another work [41] addressed the path planning for the problem of an aircraft climbing using PSO and a two-level optimization scheme, in order to improve search performance, with results showing 15% faster climbs saving 20% more fuel.

Later, in 2018, and also using PSO, Ref. [42] compared their improved PSO algorithm against other evolutionary algorithms, showing better results finding collision free and feasible paths along with minimum length and terrain roughness for a car-like mobile robot. Using an adapted version of NSGA-II, Ref. [43] was able to optimize three objectives and study the influence of the input parameters, when applying it to mobile robots on a 2D plane, obtaining fast optimization speeds and a good convergence of solutions. Also applied to mobile robotics, Ref. [44] proposed a multi-objective evolutionary algorithm, tested using five scenarios and quality metrics.

Using a combination of ant colony and PSO on different optimization levels was studied by [45], to find the best energy-efficient path for underwater vehicles on an eddy field, using uncertainty levels. An algorithm to do multi-objective path planning using PSO was proposed by [46] applied to mobile sink in wireless sensor networks, employing the concept of Pareto front to select the global and local best solutions, outperforming other algorithms in several performance metrics.

Considering aerial vehicles, Ref. [47] used a genetic algorithm to do collision-free shortest path planning, testing their proposal with and without restrictions to the path of aerial vehicles, stating that their solution can be applied to 3D environments. Another solution for multi-objective path planning for aerial vehicles was proposed by [48], combining a genetic algorithm with ant colony and using maps of height, risk, quantity, and value of sensing information. Authors used small population sizes and generations and were able to obtain dynamic environmental adaptability and high utility paths. Ref. [49] used an improved genetic algorithm to do multi-objective path planning, applied to a free-form surface milling, with three fitness functions (efficiency, energy, and carbon footprint) being converted to one single fitness function and replicating the travelling salesman problem. Another approach to multi-objective path planning using a genetic algorithm was done by [50], by using both dynamic and static 2D environments and concluding that their matrix-binary codes approach is more efficient and robust than random search algorithms.

The usage of hybrid algorithms, was detailed later in [51], applying hybrid particle-swarm optimization–modified frequency bat optimization algorithm to tackle 2D path planning. The fitness functions were collision-free path, path-smoothness, and shortest-distance, assuming some simplifications to ease the process, such as no kinematic constraints, circular obstacles, and the addition of the robot size to the obstacle size.

More recently, in 2019, Ref. [52] combined the concepts of robust optimization and the integration of multiple scenarios as a multi-objective function, in order to investigate the relation between dominance and robustness.

Using a parallel genetic approach, Ref. [53] tackled optimal path planning for single and multiple underwater vehicles, avoiding upstream currents in multi-objective optimizations by developing a new crossover operator in order to raise the diversity of the solution. Authors state two aspects when dealing with underwater vehicles: the importance of path planning tools and the usage of simulations to validate solutions before real tests.

The usage of a multi-objective firefly algorithm was proposed by [54], which saves elite particles obtained on each generation to ensure a higher ability to escape local optima results, increases the convergence speed and precision of the solution, being suitable for high-complexity multi-objective optimization problems.

## 2. Multi-Objective Optimization Applied to Underwater Glider Path-Planning

With this work, authors want to address the problem of multi-objective glider path planning using the evolutionary algorithm NSGA-II. This algorithm has been widely used for MOOP since its creation [3], becoming a standard. This algorithm is still considered a competitive candidate in many optimization challenges, and has never been used for the kind of glider path planning problems proposed in this paper.

Some basic preliminary results were obtained in the conference paper [55], as an initial exploratory proof of concept. Here the idea is extended, formally defined, and the experimental design fully developed in order to get insight into the algorithm behaviour, and obtain statistically significant conclusions.

This application problem is of special interest from the optimization point of view, due to the special vehicle operation characteristics and the complex environment uncertainties. On the other hand, the results could have many practical applications, in the form of decision support tool for multi-objective glider missions, since they are always defined as multi-purpose.

Regarding the related work previously presented, the closer approaches are [56,57]. The first one also analyses NSGA-II parameters influence in a MOPP problem, but the robot behaviour (it is not a glider) is much simpler there, and the scenario is 2D and does not include temporal variation, so building a path is not conditioned by the environment like in our case. The second work, similarly considers the use of gliders, but no simulation details are provided, and the navigation problem seems to be simplified, discarding temporal and 3D factors; additionally, the use of intermediate and target waypoints, instead of headings, introduce side effects depending on the precision ratio defined.

The main contribution of this work is two-fold: on one side, provide results in a new application problem for the NSGA-II algorithm; on the other side, contribute to the building of navigation assistance systems for glider pilots, or even autonomous navigation system, in complex missions.

### 2.1. Problem Definition

For this work, the problem of a glider that needs to navigate from a starting point to a final destination was tackled; along the path, several obstacles need to be avoided. The goal is to propose the most convenient planning taking into account the vehicle characteristics, the 3D ocean currents forecasts and the bathymetric and operational restrictions. Since ocean models produce predictions generally up to 3 days, the planning horizon has been fixed for that period too.

Two objective functions of interest have been defined, to be optimized by NSGA-II algorithm:

f(1)=Distance(Target,LastPoint)

f(2)=DistanceToObstacle(Trajectory)

f(1) function is computed as the geodesic distance between the latitude–longitude coordinates of the desired target point (Target) and the final point of the glider trajectory (LastPoint). It is a minimization objective.

f(2) function is computed as the minimal geodesic distance between the latitude–longitude coordinates of any point of the glider trajectory (Trajectory) and any of the defined obstacles. It is a maximization objective.

### 2.2. Methodology

The validation of the hypothesis was based on a series of experiments executing different variants of the optimization algorithm (through changes of the input parameters, as described on Section 3.3) over a set of selected scenarios. A significant number of independent runs were carried out to obtain statistically robust results and to give support to the conclusions.

A glider simulator took the inputs from the problem definition and environmental conditions, to produce the required outputs to be connected to the NSGA-II optimizer variants. After pre-defined stopping criteria, each algorithm returned the best individuals and the objective functions values.

The different outputs were evaluated and compared, generating different groups of results, to decide what algorithm parametrization yields better performance.

## 3. Experimental Setup

This section describes the different elements that configure the design of experimental setup: the glider simulator (used to obtain the trajectories according to the input parameters: headings, ocean forecasts, and glider displacement velocities), the scenarios (eight different scenarios in order to obtain significant data) and the algorithm variants (through a combination of parameters). Each of these elements are detailed next.

### 3.1. The Glider Simulator

A 4D (latitude, longitude, depth, and time) C-Language glider kinematic simulator was implemented, trying to reproduce, as close as possible, the real vehicle behaviour, but without introducing unnecessary computational cost that could lead to unacceptable optimization times. The simulator takes as inputs the glider displacement velocities, data about the trajectory profile, and mission parameters (start and target points, maximum and minimum depths, and number of yo-yo’s per stint, to mention some), a list of control headings to be sequentially selected after every surfacing, and the forecasted ocean currents.

This simulator has been adapted from a Matlab version that was used in previous works [13,14,15]. It has been redesigned to be able to respond to the much higher computational demands the present work requires; however, exhaustive testing has been performed in order to guarantee that both simulators produce the same results under the same input conditions.

The glider control configuration was set to include 10 stints composed by two yo-yo’s each, defining the inflection range of a maximum depth of 1060 m and a minimum of 10 m. The vehicle was commanded to stay at the surface for 20 min before the next dive. The nominal velocities were configured to model the performance of the Slocum G2 glider, also incorporating some manoeuvrability restrictions. Figure 1 shows an example of 3D glider trajectory, illustrating the vehicle inflections, surface drifting, and tidal-current effects.

### 3.2. Simulation Scenarios

**Figure 2:**
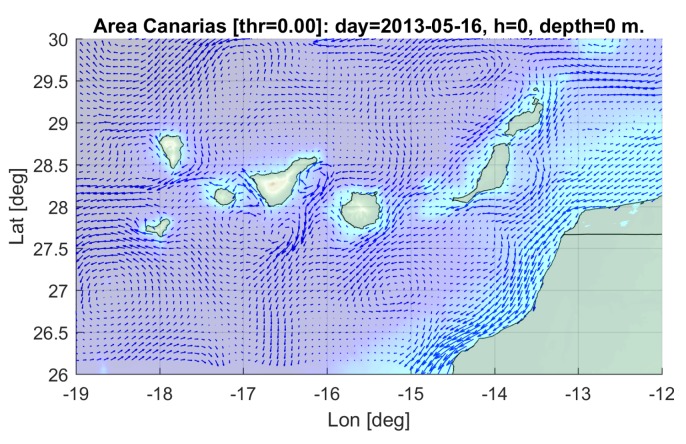
Surface currents of the area of simulation (Canary Islands, NE Atlantic).

The Canary Islands sea area was selected for the simulation, due to the special characteristics found in this zone, with the presence of a great variety of oceanographic structures of interest (eddies, upwelling cells, fronts, etc.). The area covered from −12 to −19${}^{\circ }$ East and 26 to 30${}^{\circ }$ North, between 16 and 18 May 2013, a period showing highly dynamic ocean currents, as can be observed in Figure 2.

Ocean circulation forecasting maps (U and V variables) for the glider path simulator are read from MERCATOR-IBI [56], with a spatial resolution of 1/12${}^{\circ }$ and daily outputs, with 3D data from surface down to 5000 m depth. Other ocean forecasts can also be used, but the decision of using this particular product is because its spatial resolution (approximately 2 km).

**Figure 3:**
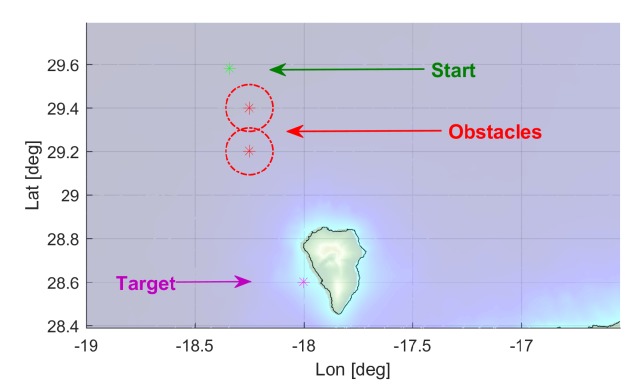
Scenario elements example: start point, target, and obstacle’s locations.

To test this system and its robustness, a set of eight scenarios was defined around the islands. Each scenario, as shown in Figure 3, consist in a start location (green), two obstacles with a safety ratio (red), and a target location (purple). The factors considered were the ocean currents average direction (favourable/against) and variability (stable/unstable), obstacle configuration (overlapping/separated), and bathymetric restrictions.

Four scenarios with different trajectory directions (North–South, South–North, East–West, and West–East), are configured according to Table 1 for overlapping obstacles and Table 2 for same trajectories’ direction but with separated obstacles. In all cases, the obstacle safety radius has been fixed to 12 km. The distance between the starting point and the target has been selected so it’s not reachable in the 3 days planning horizon.

**Figure 4:**
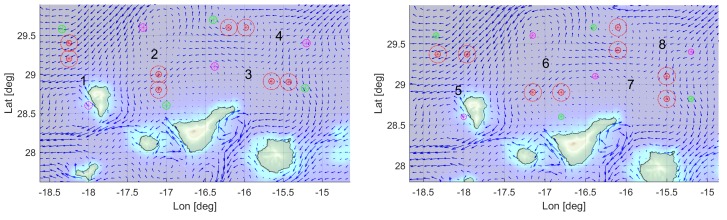
Areas of simulation for overlapping (**left**) and separated (**right**) obstacles, with surface ocean current directions.

Images in Figure 4 show the location of the different elements integrating the corresponding overlapping and separated scenarios in the map.

Regarding the average direction of the 3D ocean currents, scenarios 1, 3, 5, and 7 correspond to favourable currents, while scenarios 2, 4, 6, and 8 represent against currents. With respect to the variability factor, the scenarios 2, 3, 6, and 7 exhibit lower stability in the currents temporal evolution, compared to scenarios 1, 4, 5, and 8, that are more stable.

### 3.3. NSGA-II Configuration

As previously commented, NSGA-II algorithm is considered a standard for solving MOOP. Briefly, NSGA-II starts by initializing a population, which is evaluated and ranked based on non-dominated sorting and the crowding distance operator. After, it runs a main loop composed by selection, crossover, and mutation operators, to generate an offspring which is evaluated through the fitness functions. Next, it combines and evaluates the parent and the newly obtained offspring and selects some individuals by their rank. This loop runs until a stopping criterion is met, and the final population is reported. A more detailed explanation and the respective pseudo-code is presented in the detailed description of the algorithm [3,23].

The chromosome is constituted by a total of 10 real variables, representing different headings to the glider at every surfacing, and set to be kept by the vehicle along each underwater stint. Without loss of generality, an incremental heading change codification is chosen instead of absolute waypoint coordinates to avoid undesirable target hit conditions and waypoint transition effects. Minimum and maximum values of all the genes are −180 and +180 sexagesimal degrees, respectively.

The objective of these experiments is to find the best parameter combination through the execution of multiple optimizations, analyse and present the best results according to statistical tests, and interpret those results in the context of glider path planning.

A total of 27 different configurations were considered for the NSGA-II, combining three values of mutation probability (0.05, 0.1, 0.15) around the $\frac{1}{n}$ rule [57], with *n* = 10 in our case, and crossover probability (1, 0.9, 0.8), with population size (40, 100, and 160) for 500, 200, and 125 generations, respectively. To guarantee a fair comparison, the same total number of 20,000 fitness evaluations (FES) was set as stopping criterion. All parameter value combinations are presented in Table 3.

The glider simulator was coupled with NSGA-II, that was configured combining values of multiple parameters (Table 3). Every configuration of each case is repeated for 51 independent random executions in order to obtain statistically significant results for the evaluation of the performance of the meta-heuristic optimization. The coupled system output for every run is a list composed by the vehicle position on a 4D representation (Latitude, Longitude, and Depth over time), according to the provided headings and ocean circulation data.

With the configurations presented, and using a regular laptop (i7 6700HQ, 16 GB RAM, running Linux Ubuntu 18.04) executing 4 optimizations simultaneously, each one of the 8 cases (27 configurations × 51 random seed values, each) was performed in about one hour.

As the version of NSGA-II used and the simulator are written in the C-Language, the requirements to run these experiments are, essentially, a computer with a C compiler and the netCDF libraries (in order to read the ocean forecast data). The amount of time needed to optimize will depend on the hardware specifications of the machine used.

## 4. Results

Departing from the configurations and scenarios described in the previous section, a complete set of experiments were carried out. The respective results are presented next, and discussed in Section 5.

The hypervolume (HV) indicator [58] will be used to evaluate the results as indicator of multi-objective relative quality, measuring the n-dimensional volume with respect to a pre-defined reference point [59]. The HV values are used to rank solutions and assess global optimization convergence.

**Figure 5:**
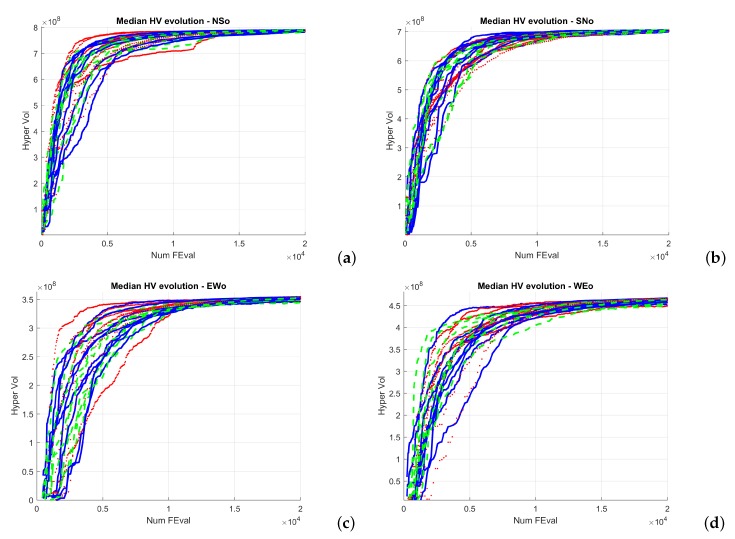
Hypervolume (HV) median convergence for scenarios 1–4 after 20,000 executions fitness evaluations (FES): (**a**) Scenario 1 (N–S); (**b**) Scenario 2 (S–N); (**c**) Scenario 3 (E–W); (**d**) Scenario 4 (W–E).

**Figure 6:**
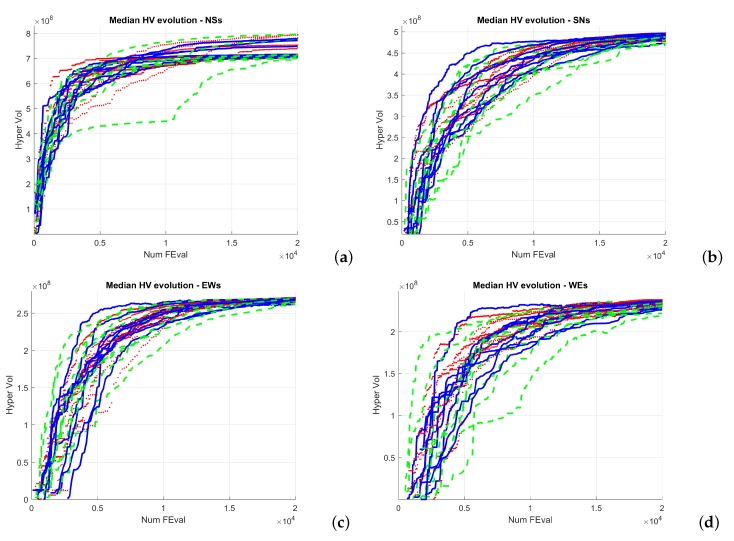
HV median convergence for scenarios 5–8 after 20,000 executions FES: (**a**) Scenario 5 (N–S); (**b**) Scenario 6 (S–N); (**c**) Scenario 7 (E–W); (**d**) Scenario 8 (W–E).

Figure 5 and Figure 6 show the evolution of the Hypervolume for the median execution of every 27 configurations, for overlapping and separated obstacles scenarios, respectively. The reference points used for HV computation were 10 km and 90 km (80 km for scenarios 6 and 8).

Hypervolume evolution graphs show that the optimization process has reached a stable value, so no significant improvement is expected by increasing the number of generations.

### 4.1. Individual Scenario Analysis

This subsection will present results obtained for every scenario individually, using the final HV value as a comparison measure.

First, a Friedman statistical analysis is performed to test the null hypothesis that all parameter combinations produce similar results. In all cases, the computed *p*-value for the statistic (chi-square distribution with 26 degrees of freedom) is small, so the null hypothesis can be rejected. Table 4 compiles the Friedman test results.

The average rankings of every configuration and scenario are compiled in Table 5.

**Figure 7:**
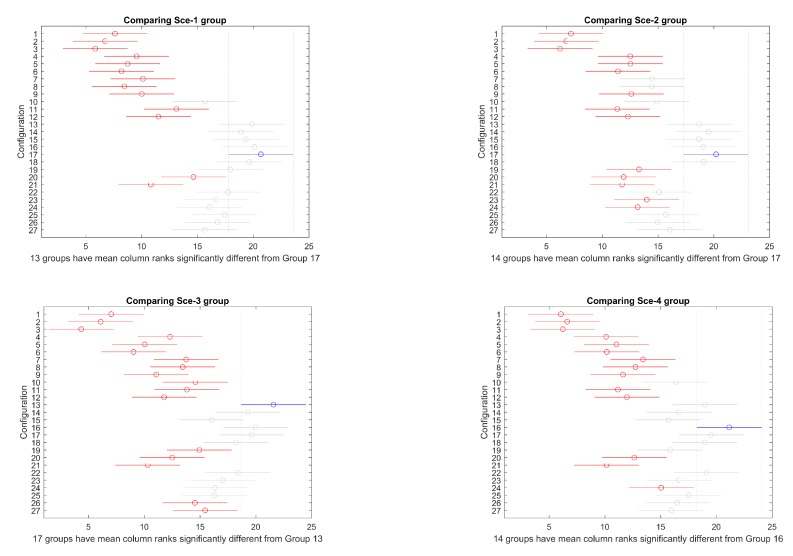
Mult–compare test of (Tukey–Kramer) results for scenarios 1–4.

**Figure 8:**
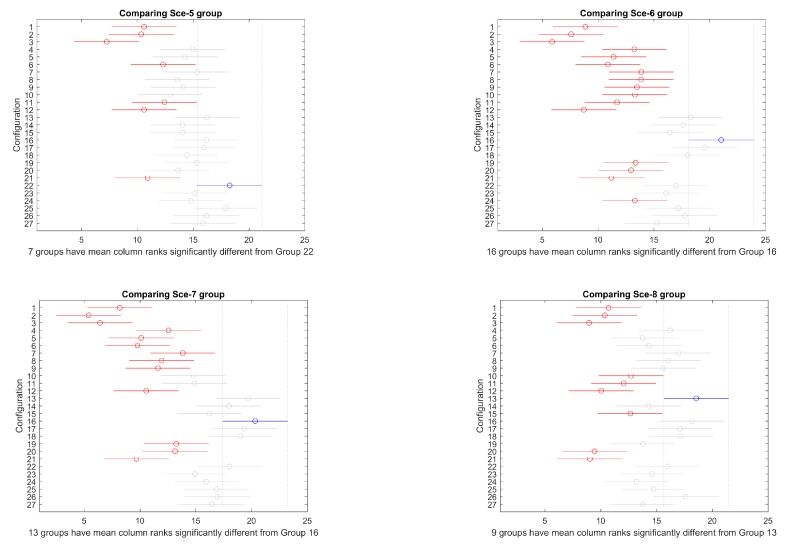
Mult–compare test of (Tukey–Kramer) results for scenarios 5–8.

Then, a mult-compare test is performed to identify, more specifically, the differences among configurations. Figure 7 and Figure 8 present the results graphically for overlapped and separated scenarios, respectively. In every figure, the best configuration is marked in blue, the ones that produce significantly worse results are marked in red, and the ones that do not perform significantly worse are marked in grey. Critical values are based on Tukey–Kramer method.

**Figure 9:**
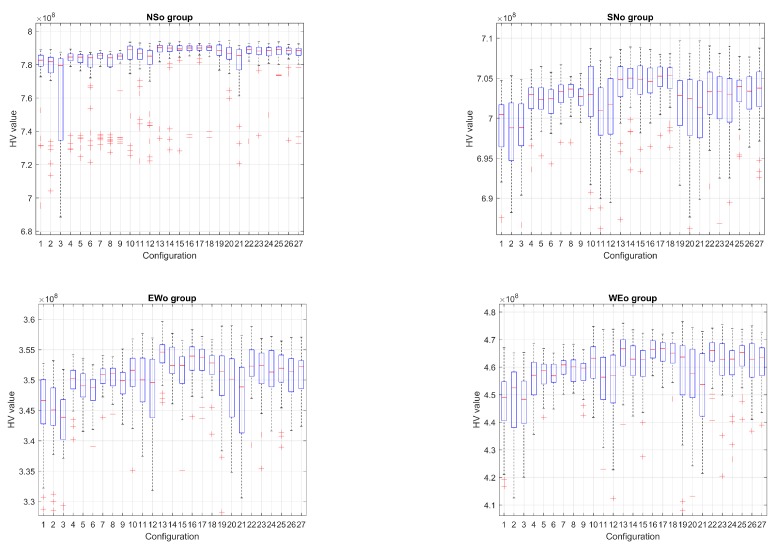
Box plots for scenarios 1–4 (overlapping obstacles.)

**Figure 10:**
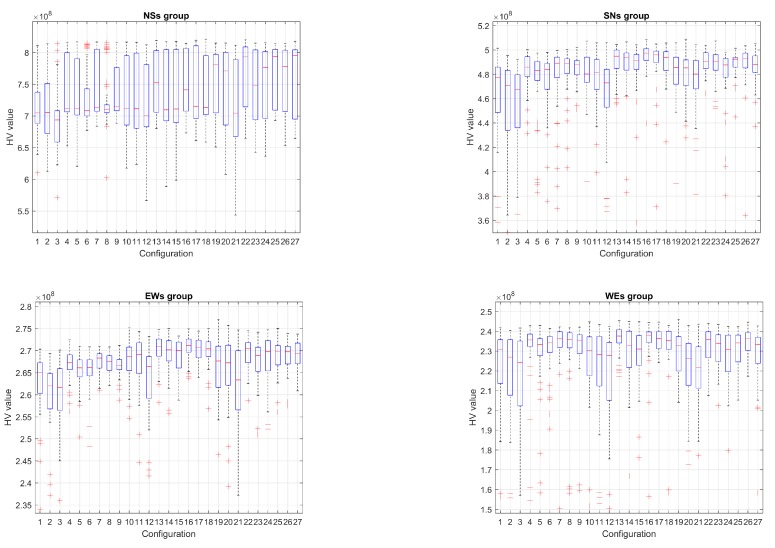
Box plots for scenarios 5–8 (separated obstacles.)

The corresponding box plot representations for the final HV values (51 executions × 27 configurations) can be observed in Figure 9 and Figure 10 for every scenario. In order to get a better view, the axis have been zoomed around the upper and lower adjacent values, so distant outliers are not shown.

**Figure 11:**
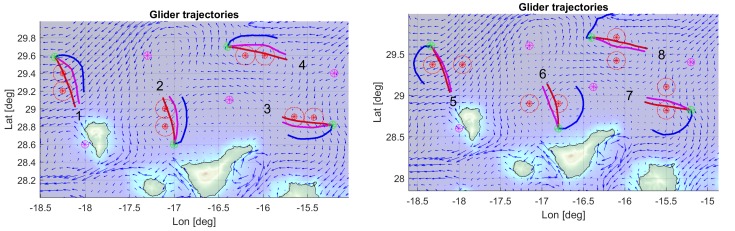
Examples of optimized trajectories for overlapping (**left**) and separated (**right**) obstacles scenarios.

Before going into more detailed analysis, a global perspective of the optimization results is presented in the images depicted in Figure 11, showing some selected glider trajectories resulting from the multi-objective optimization process. Although the trajectories are represented here as 2D, as previously stated, they are in fact 4D as the vehicle evolves in time across X (Longitude), Y (Latitude), and Z (Depth) coordinates during the three-day mission, from start towards the target point (pattern on Figure 1).

In general, all comparatives follow a similar structure. Scenarios 1 and 8 will be selected as an example to have a closer look to the optimization process.

#### 4.1.1. Scenario 1 (NS Overlapped)

Configuration 17, corresponding to PS 100, CP 0.9, and MP 0.15, produces the best result in this scenario, according to Figure 7.

From a post-hoc procedure [60], Table 6 gives the Bergmann–Hommel test adjusted *p*-values compared to case 17. Considering a critical value of α=0.05, the first 21 configurations perform significantly worse than case 17.

The more conservative Bonferroni test produces the adjusted *p*-values presented in Table 7. According to the critical value α=0.05/27, the first 12 configurations perform significantly worse than case 17.

**Figure 12:**
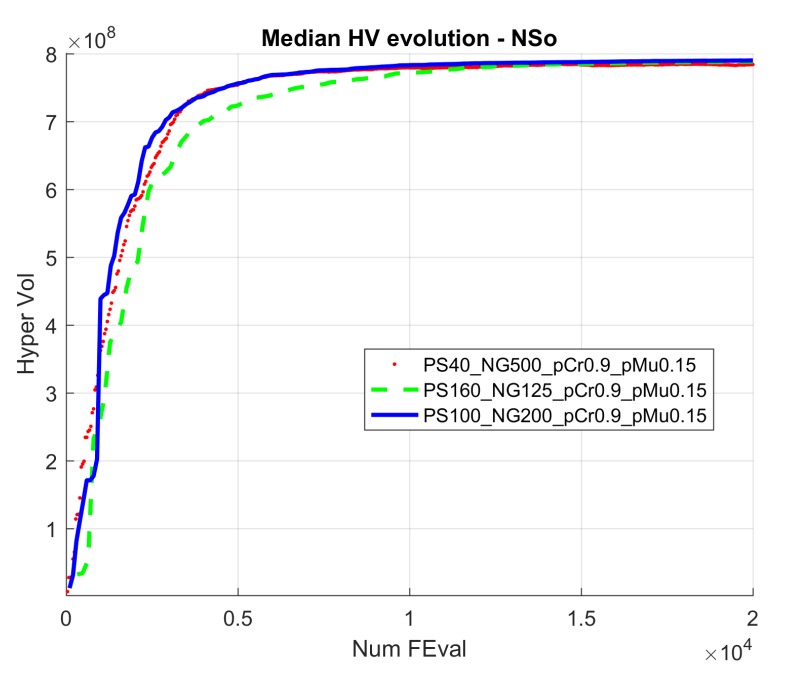
Hypervolume evolution for configurations 17, 9, and 26 (median run) in scenario 1.

Figure 12 shows the evolution of the median run hypervolume for configuration 17 and also for configurations 9 and 26, that have same probability values for population sizes 40 and 100.

**Figure 13:**
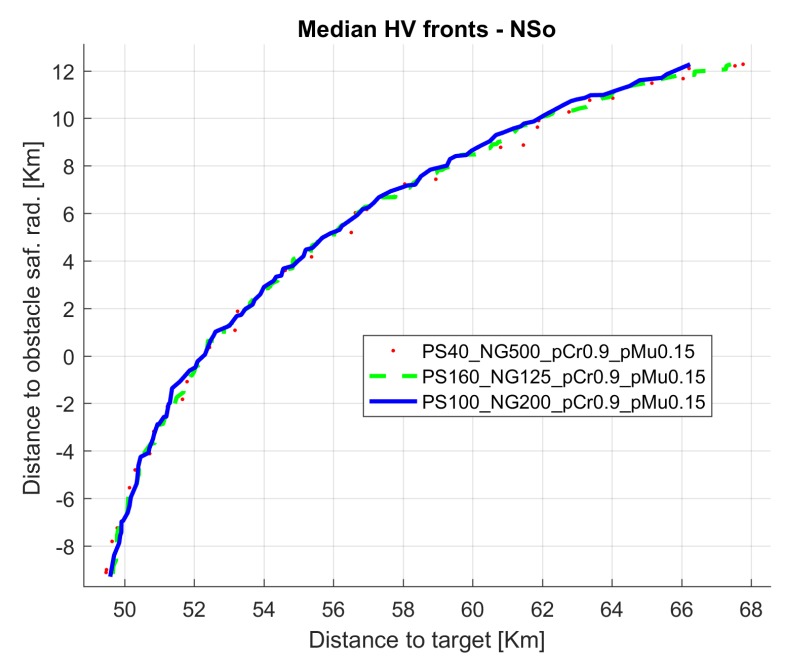
Non dominated fronts for configurations 17, 9, and 26 (median run) in scenario 1.

Figure 13 presents the non dominated front for median run for the same 17, 9, and 26 cases. The horizontal axis presents the objective function distance to target (f(1)) and the vertical axis presents the distance to obstacle safety radius (f(2)−safRad); negative values indicate that the glider trajectory crossed the safety radius of the obstacles.

**Figure 14:**
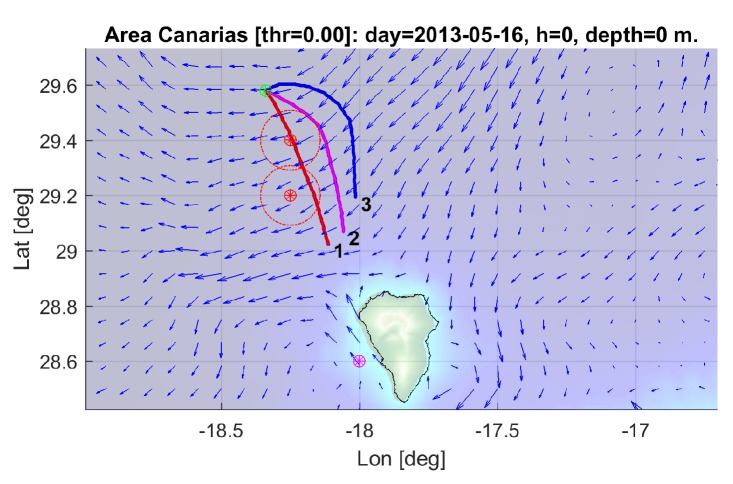
Selected trajectories for scenario 1 (1: best distance to end; 2: best distance to end and obstacle; 3: best distance to obstacle.)

**Figure 15:**
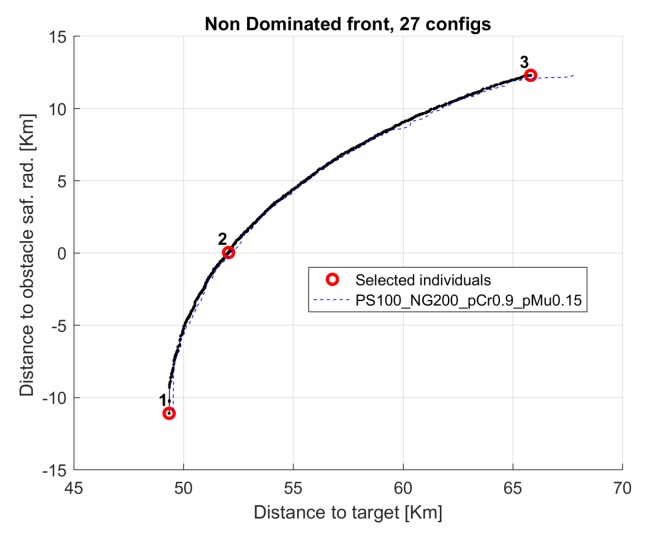
Selected individuals for scenario 1.

The results of the multi-objective optimization are illustrated in Figure 14, where three trajectories corresponding to selected individuals from the final accumulated non dominated front optimization are shown (see Figure 15). Trajectories 1 and 3 correspond to extreme unpractical solutions, and are shown just to represent one sided optimization for distance (trajectory 1) and for safety (trajectory 3). Trajectory 2 represents a more reasonable solution, in this case, selected as the first one that verifies the safety radius.

Table 8 details the optimized variable values (incremental headings) for the individuals and trajectories previously illustrated for scenario 1.

#### 4.1.2. Scenario 8 (WE Separated)

In this case, the best configuration according to Figure 5 is 13, corresponding to PS 100, CP 1.0, and MP 0.10.

The Bergmann–Hommel test adjusted *p*-values compared to case 13 are shown in Table 9 gives. The first 18 configurations perform significantly worse than case 13, for the remaining 8, the null hypothesis (critical value of α=0.05) of having similar performance.

Bonferroni test produces the adjusted p-values presented in Table 10. According to the critical value α=0.05/27, the first six configurations perform significantly worse than case 13.

**Figure 16:**
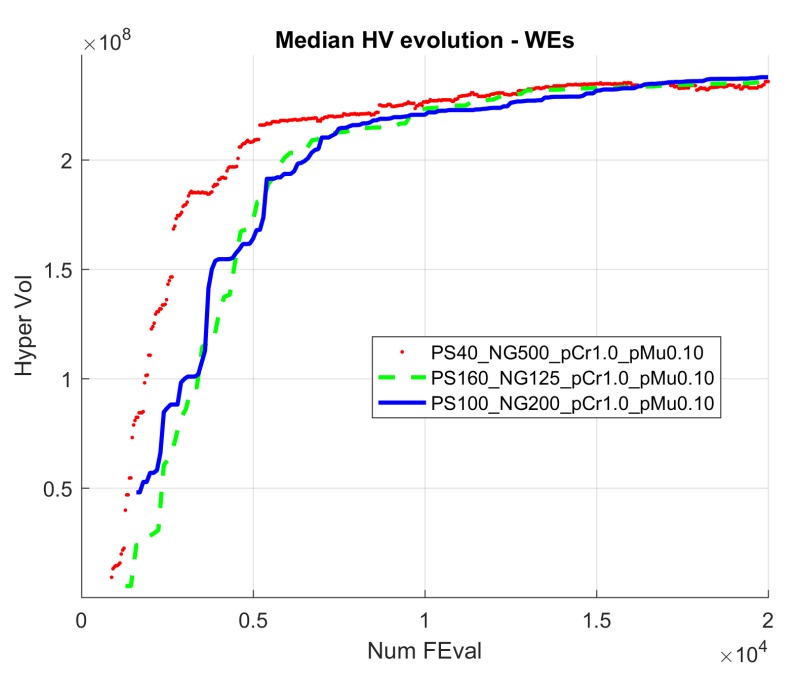
Hypervolume evolution for configurations 13, 4, and 22 (median run) in scenario 8.

Figure 16 shows the evolution of the median run HV for configuration 13 and also for configurations 4 and 22, sharing the same probability values for population sizes 40 and 160.

**Figure 17:**
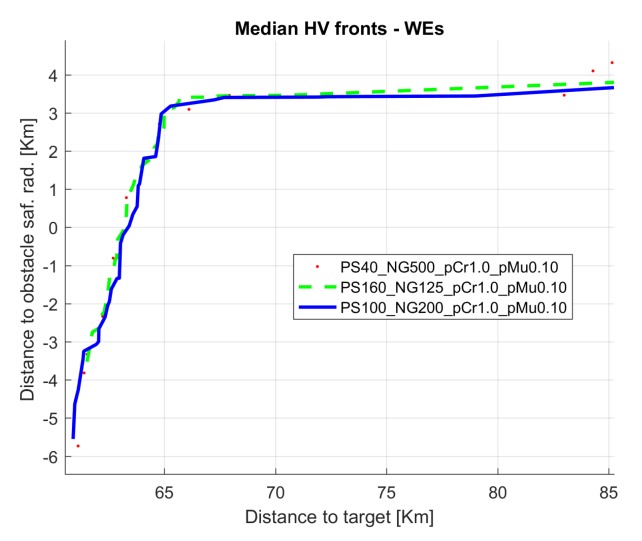
Non dominated fronts for configurations 13, 4, and 22 (median run) in scenario 8.

Figure 17 presents the non dominated front for median run for the same configurations (13, 4, and 22).

**Figure 18:**
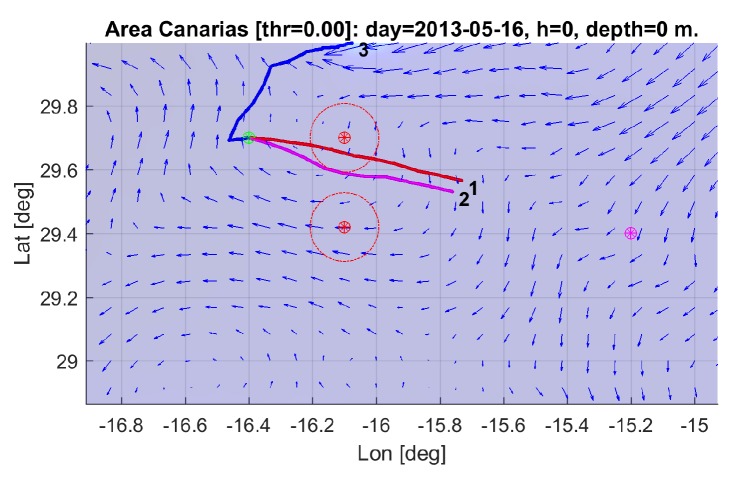
Selected trajectories for scenario 8 (1: best distance to end; 2: best distance to end, and obstacle; 3: best distance to obstacle.)

**Figure 19:**
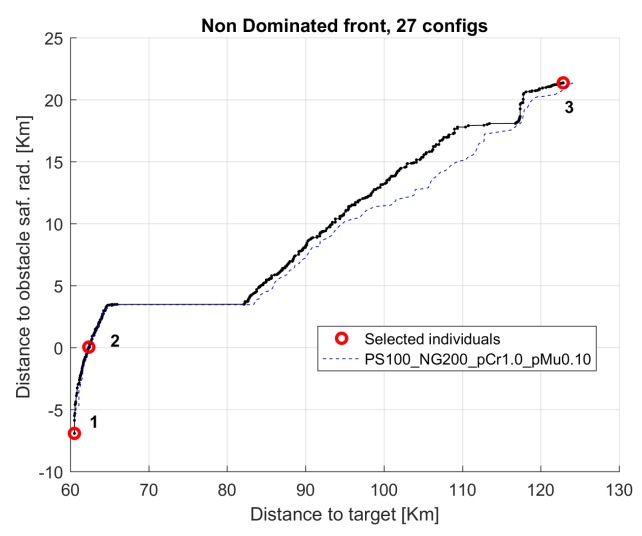
Selected individuals for scenario 8.

The results of the multi-objective optimization are illustrated in Figure 18, where three trajectories corresponding to individuals from the final accumulated non dominated front optimization (Figure 19) are shown. As explained previously, trajectories 1 and 3 are presented for illustrative purposes, while trajectory 2 shows the closest to safety radius solution.

Compared to scenario 1, the optimization process is more restricted here, and the level of significant differences between algorithm variants results is reduced.

The optimized variable values (incremental headings) corresponding to the individuals and trajectories previously illustrated for this scenario are detailed in Table 11.

### 4.2. Grouped Scenario Analysis

#### 4.2.1. Overlapped vs. Separated Obstacles

**Figure 20:**
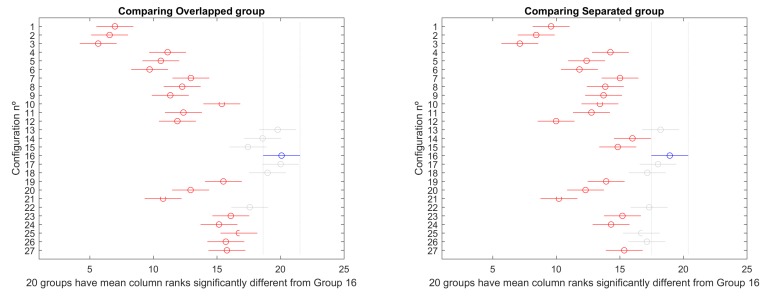
Mult-compare (Tukey–Kramer) results for overlapping scenarios 1–4 (**left**) and separated scenarios 5–8 (**right**).

Selecting the object configuration as grouping factor, overlapping scenarios 1, 2, 3, and 4, on one side, and scenarios 5, 6, 7, and 8, on the other, have been compared. The mult-compare graphs are shown in Figure 20. Configuration 16 is the top ranked, producing better statistically significant results for all other alternatives, except 13, 14, 15, 17, 18, and 22 in overlapped group, and 13, 17, 18, 22, 25, and 26 for separated group.

#### 4.2.2. Favourable vs. against Ocean Currents

**Figure 21:**
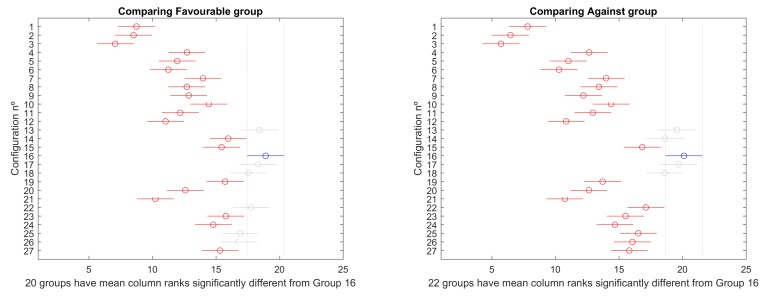
Mult-compare (Tukey–Kramer) results for favourable currents scenarios 1, 4, 5, and 8 (**left**) and against scenarios 2, 3, 6, and 7 (**right**).

Selecting the ocean currents direction as grouping factor, favourable currents scenarios 1, 2, 3, and 4, on one side, and scenarios 5, 6, 7, and 8, on the other, have been compared. The mult-compare graphs are shown in Figure 21. Configuration 16 is the top ranked, producing better statistically significant results for all other alternatives, except 13, 17, 18, 22, 25, and 26 in favourable group, and 13, 14, 17, and 18 for against group.

### 4.3. Overall Comparison

**Figure 22:**
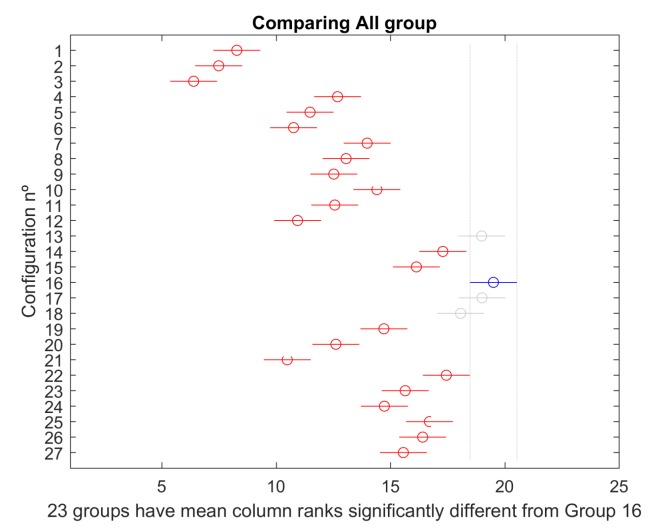
Comparison of all scenarios.

Considering all group simulations combined, the mult-compare test produces the results presented in Figure 22. Again, configuration 16 dominates significantly all other alternatives except 13, 17, and 18.

## 5. Discussion

From the analysis of the HV evolution (Figure 5 and Figure 6), the trend is to reach a steady state. This means that the resulting trajectories cannot be much more improved without prejudice of the global solutions. In practical terms, obtained trajectories will not have significant improvements regarding the distance to the target point versus the distance to the obstacles even if the optimization process kept being executed.

Considering all the configurations of both cases, configuration 16 (PS 100, NG 200, CP 1, MP 0.15) provided the best global results, closely followed by 13 (PS 100, NG 200, CP 1.0, MP 0.10) and 17 (PS 100, NG 200, CP 0.9, MP 0.15) (Figure 22). The population size of 100 is a good balance for exploration–exploitation equilibrium of the search in these 10 variable tested scenarios. It provides a faster convergence than the 160 population size case, while a highly elitist algorithm as NSGA-II favours a high crossover rate (1.0) and slightly higher mutation rate (0.15) than the general rule 1/n (0.1), enhancing the population diversity by those operators. Second and third configurations also keep either the crossover rate of 1.0 with mutation rate of 0.1 or a slightly lower crossover rate (0.9) with mutation rate of (0.15), confirming the appropriateness of the operators.

As for the parameter values, when keeping PS and MP, results worsen with the decrease of CP, meaning that resulting trajectories, overall will present worse results for the fitness functions, either by ending far from the target point and passing too close to the obstacles, two undesirable situations.

If CP is kept constant, results get better with the increasing of MP, except for PS 160, CP 0.8, and MP 0.15, meaning better balanced distances both to target and to the obstacles. Also, increasing the MP and decreasing CP for each PS results in less dispersion of the results except for PS 50 and MP 0.1.

Considering the cases of separated versus overlapping obstacles, results show a bigger dispersion on the overlapping cases. Also, although configuration 16 (PS 100) presented the best results on both cases, on overlapping cases some configurations with PS 160 presented closer results to 16 than on the separated case, on which is evident that cases with PS 100 (except 22) are the best ones.

Another aspect that impacts the results is the direction of ocean currents. In scenarios with favourable currents, cases with PS 160 presented results closer to configuration 16, opposite to unfavourable currents, on which cases with PS 100 are closer to configuration 16.

Specific scenario analysis—e.g., scenarios 1 and 8 in this paper—could be performed to select the most convenient configuration depending on the particular environmental characteristics, instead of applying the global solution. The results of the post-hoc procedures indicate that there is evidence of statistically significant advantages among the different configurations. Particularly, compared to Bonferroni’s the more powerful Bergmann–Hommel procedure is able to identify a higher number of cases surpassed by the best performing combination; 9 more cases in scenario 1 (Table 6 and Table 7) and 12 in scenario 8 (Table 9 and Table 10).

Apart from pure numerical evaluation, the algorithm configuration selection could also depend on additional factors such as available computational resources or time restrictions.

## 6. Conclusions and Future Work

Overall, a useful methodology/tool was built and can be applied to multi-objective underwater glider path planning, obtaining a set of non-dominated solutions simultaneously minimizing the distance to target and maximizing the minimum distance to one or more obstacles. Multiple simulations were performed in the geographic region of Canary Islands, demonstrating the suitability of the methodology. Those tests included favourable, side, and against ocean currents, different obstacle locations and different start and target points, all of them possible situations that can occur during a glider mission.

The system described here can now be easily configured and tuned to be used as a planning tool for glider pilots, to help determine, together with their expertise and knowledge, which are the best trajectories to pursue during a specific mission. Obviously, the results are conditioned by the quality of the ocean currents data and/or forecast, due to the uncertainty associated to forecasts. In any case, higher skill rank predictors are continuously proposed, with better spatial and temporal resolution characteristics.

The proposed methodology was able to produce satisfactory trajectories using the genetic algorithm NSGA-II. It is worth mentioning that the optimizations include well spread solutions that go from one extreme to another. This means that solutions include results that, in this case, crossed the safety radius to obtain a better distance to target point (through a straight line) or move away from the safety radius, increasing safety at the expense of worse target distances.

From the results obtained and discussed previously, it is possible to state that the described system, through the usage of the genetic based evolutionary algorithm NSGA-II, is able to produce usable paths, being the best ones obtained using configuration 16 (PS 100 NG 200 CP 1.0, and MP 0.15) for all simulated cases.

The main contributions seek with this work were also achieved: NSGA-II can be used to optimize multi-objective glider path planning, an innovative and challenging engineering problem, never addressed this way. The best parameter combination was found for the designed scenarios (configuration 16), but the system can be adjusted to other locations, to benefit control teams, in need of valuable information for critical decision making situations, potentially extending the vehicle operational potential and thus increasing the mission productivity.

Future works include considering aspects like battery consumption to accomplish the mission, areas of interest to intensify sampling or marine traffic that represent real obstacles to glider navigation. These aspects can be included as additional fitness functions with more parameters to be taken into account. Also, a comparison with other evolutionary algorithms could be done, to evaluate the complexity and performance of the described system against other optimization techniques, on a more extensive work. The usage of very high-resolution forecasts, with more outputs per day and additional oceanographic/atmospheric variables can also be an interesting possibility to consider, as the solutions are as accurate as the accuracy of the forecasts provided. With all these aspects to be considered on a future work, as they increase the computational costs, the optimizations will need to be executed on a computational cluster, in order to run even more optimizations simultaneously. Also, using a GPGPU (general-purpose graphics processing unit) could be a relevant improvement, due to their ability to do massive parallelism and speed up the optimization process. Progressively moving this infrastructure to standard platforms like ROS is also planned to ease sharing results, provided that authors are able to keep optimization times inside practical levels. As a final remark, it would be interesting to validate the results obtained with real glider missions; something complicated in this moment since it would require exclusive multi glider use for long periods.

## Figures and Tables

**Table 1:** Scenarios coordinates (${}^{\circ }$ N; ${}^{\circ }$ E) of the start point, target, and overlapping obstacles for the four directions.

Scen.Id.	Direction	Start (${}^{\circ }$ N; ${}^{\circ }$ E)	Target (${}^{\circ }$ N; ${}^{\circ }$ E)	Obstacle 1 (${}^{\circ }$ N; ${}^{\circ }$ E)	Obstacle 2 (${}^{\circ }$ N; ${}^{\circ }$ E)
1 (NSo)	North-South	29.60; −18.34	28.60; −18.00	29.40; −18.25	29.20; −18.25
2 (SNo)	South-North	28.60; −17.00	29.60; −17.30	28.80; −17.10	29.00; −17.10
3 (EWo)	East-West	28.82; −15.22	29.10; −16.38	28.90; −15.43	28.91; −15.65
4 (WEo)	West-East	29.70; −16.40	29.40; −15.20	29.60; −16.20	29.60; −15.98

**Table 2:** Scenarios coordinates (${}^{\circ }$ N; ${}^{\circ }$ E) of the start point, target, and separated obstacles for the four directions.

Scen.Id.	Direction	Start (${}^{\circ }$ N; ${}^{\circ }$ E)	Target (${}^{\circ }$ N; ${}^{\circ }$ E)	Obstacle 1 (${}^{\circ }$ N; ${}^{\circ }$ E)	Obstacle 2 (${}^{\circ }$ N; ${}^{\circ }$ E)
5 (NSs)	North-South	29.60; −18.34	28.60; −18.00	29.37; −18.32	29.37: −17.96
6 (SNs)	South-North	28.60; −16.80	29.60; −17.15	28.90; −16.80	28.90; −17.15
7 (EWs)	East-West	28.80; −15.20	29.10; −16.40	29.10; −15.50	28.82; −15.50
8 (WEs)	West-East	29.70; −16.40	29.40; −15.20	29.70; −16.10	29.42; −16.10

**Table 3:** Configurations tested in the experiments, varying in population size (PS), number of generations (NG), crossover probability (CP), and mutation probability (MP).

N	PS	NG	CP	MP
1	40	500	1.0	0.05
2	40	500	0.9	0.05
3	40	500	0.8	0.05
4	40	500	1.0	0.10
5	40	500	0.9	0.10
6	40	500	0.8	0.10
7	40	500	1.0	0.15
8	40	500	0.9	0.15
9	40	500	0.8	0.15
10	100	200	1.0	0.05
11	100	200	0.9	0.05
12	100	200	0.8	0.05
13	100	200	1.0	0.10
14	100	200	0.9	0.10
15	100	200	0.8	0.10
16	100	200	1.0	0.15
17	100	200	0.9	0.15
18	100	200	0.8	0.15
19	160	125	1.0	0.05
20	160	125	0.9	0.05
21	160	125	0.8	0.05
22	160	125	1.0	0.10
23	160	125	0.9	0.10
24	160	125	0.8	0.10
25	160	125	1.0	0.15
26	160	125	0.9	0.15
27	160	125	0.8	0.15

**Table 4:** Friedman test results for every scenario (1–4: overlapping obstacles; 5–8 separated obstacles).

Scenario	1	2	3	4	5	6	7	8
***p*-value**	2.67046 × $10^{-84}$	6.47715 × $10^{-48}$	8.68828 × $10^{-69}$	7.36995 × $10^{-63}$	7.7676 × $10^{-16}$	5.1261 × $10^{-48}$	1.01802 × $10^{-58}$	7.9773 × $10^{-24}$

**Table 5:** Average ranking for every scenario.

C_1	AvR_1	C_2	AvR_2	C_3	AvR_3	C_4	AvR_4	C_5	AvR_5	C_6	AvR_6	C_7	AvR_7	C_8	AvR_8
17	7.31	17	7.78	13	6.41	16	6.84	22	9.73	16	6.96	16	7.67	13	9.43
16	7.88	14	8.45	16	8.00	17	8.47	25	10.06	17	8.43	13	8.29	16	9.84
13	8.12	18	8.90	17	8.37	22	8.88	13	11.75	13	9.69	17	8.63	26	10.39
18	8.35	16	8.92	14	8.69	18	9.02	16	11.80	18	9.96	18	9.00	18	10.84
15	8.65	13	9.31	22	9.61	13	9.04	26	11.82	26	10.22	22	9.98	17	10.88
14	9.10	15	9.35	18	9.78	25	10.49	17	12.04	14	10.35	14	10.02	7	11.02
19	10.04	27	11.94	23	10.96	23	11.35	27	12.10	25	10.80	26	11.06	4	11.78
22	10.25	25	12.29	24	11.65	14	11.37	7	12.63	22	11.04	25	11.12	8	11.94
25	10.57	22	12.90	25	11.73	26	11.51	19	12.69	15	11.57	27	11.55	22	12.02
26	11.20	26	13.04	15	11.96	10	11.63	23	12.80	23	11.90	15	11.78	9	12.37
23	11.31	10	13.10	27	12.53	27	12.04	4	13.00	27	12.73	24	12.06	25	13.25
24	11.92	7	13.51	19	13.06	19	12.16	24	13.20	7	14.12	23	13.06	23	13.41
10	12.31	8	13.57	10	13.41	15	12.27	18	13.55	8	14.14	11	13.12	6	13.67
27	12.33	23	14.00	26	13.45	24	12.94	5	13.73	9	14.51	10	13.24	14	13.69
20	13.35	19	14.71	11	14.18	7	14.57	9	13.86	19	14.63	7	14.16	5	14.24
11	14.88	24	14.82	7	14.24	8	15.25	14	13.96	10	14.71	19	14.75	19	14.24
12	16.49	9	15.39	8	14.55	20	15.35	15	13.96	24	14.71	20	14.84	27	14.24
21	17.18	5	15.47	20	15.49	12	16.02	20	14.35	4	14.75	4	15.43	24	14.78
7	17.88	4	15.49	4	15.69	9	16.37	8	14.43	20	15.04	8	16.06	10	15.27
9	17.98	12	15.71	12	16.22	11	16.82	10	15.04	11	16.29	9	16.37	15	15.33
4	18.45	20	16.08	9	16.92	5	16.96	11	15.57	5	16.61	12	17.43	11	15.94
5	19.25	21	16.22	21	17.69	6	17.82	6	15.69	21	16.80	5	17.88	1	17.29
8	19.55	6	16.59	5	17.96	21	17.84	21	17.08	6	17.12	6	18.22	2	17.63
6	19.80	11	16.65	6	18.96	4	17.88	1	17.39	1	19.14	21	18.31	12	17.94
1	20.39	1	20.80	1	20.96	2	21.37	12	17.39	12	19.27	1	19.80	20	18.55
2	21.27	2	21.22	2	21.90	3	21.76	2	17.65	2	20.41	3	21.57	21	18.96
3	22.16	3	21.78	3	23.65	1	21.94	3	20.75	3	22.12	2	22.61	3	19.04

**Table 6:** Adjusted *p*-values Bergmann–Hommel procedure for scenario 1 (ref. c17).

Rank	Case	P Bergmann–Hommel
1	C3	9.38 × $10^{-20}$
2	C2	1.64 × $10^{-17}$
3	C1	2.10 × $10^{-15}$
4	C6	4.41 × $10^{-14}$
5	C8	1.54 × $10^{-13}$
6	C5	6.36 × $10^{-13}$
7	C4	2.76 × $10^{-11}$
8	C9	2.07 × $10^{-10}$
9	C7	3.18 × $10^{-10}$
10	C21	5.95 × $10^{-09}$
11	C12	8.44 × $10^{-08}$
12	C11	2.20 × $10^{-05}$
13	C20	1.70 × $10^{-03}$
14	C27	1.68 × $10^{-02}$
15	C10	1.76 × $10^{-02}$
16	C24	3.71 × $10^{-02}$
17	C23	9.83 × $10^{-02}$
18	C26	1.21 × $10^{-02}$
19	C25	2.68 × $10^{-02}$
20	C22	3.67 × $10^{-02}$
21	C19	4.97 × $10^{-02}$
22	C13	7.17 × $10^{-02}$
22	C14	7.17 × $10^{-02}$
22	C15	7.17 × $10^{-02}$
22	C16	7.17 × $10^{-02}$
22	C18	7.17 × $10^{-02}$

**Table 7:** Adjusted *p*–values Bonferroni procedure for α=0.05/27 (scenario 1)—best case C17.

Rank	Case	P Bonferroni
1	C3	1.267427 × $10^{-18}$
2	C2	2.305458 × $10^{-16}$
3	C1	3.071510 × $10^{-14}$
4	C6	6.740886 × $10^{-13}$
5	C8	2.462380 × $10^{-12}$
6	C5	1.063101 × $10^{-11}$
7	C4	4.858586 × $10^{-10}$
8	C9	4.040082 × $10^{-09}$
9	C7	6.211904 × $10^{-09}$
10	C21	1.229053 × $10^{-07}$
11	C12	1.852669 × $10^{-06}$
12	C11	5.160795 × $10^{-04}$
13	C20	4.279908 × $10^{-02}$
14	C27	4.933295 × $10^{-01}$
15	C10	5.150743 × $10^{-01}$
16	C13	1.000000 × $10^{-00}$
16	C14	1.000000 × $10^{-00}$
16	C15	1.000000 × $10^{-00}$
16	C16	1.000000 × $10^{-00}$
16	C18	1.000000 × $10^{-00}$
16	C19	1.000000 × $10^{-00}$
16	C22	1.000000 × $10^{-00}$
16	C23	1.000000 × $10^{-00}$
16	C24	1.000000 × $10^{-00}$
16	C25	1.000000 × $10^{-00}$
16	C26	1.000000 × $10^{-00}$

**Table 8:** Optimized glider headings for scenario 1.

Headings	1	2	3	4	5	6	7	8	9	10
**Indiv-1**	138.017	6.661	−0.024	6.078	1.297	−0.529	0.009	6.938	1.176	1.638
**Indiv-2**	111.295	5.978	2.600	6.146	27.866	4.893	1.092	9.610	−1.959	−2.604
**Indiv-3**	62.607	19.845	7.893	10.632	16.704	19.213	29.566	5.705	3.118	−0.876

**Table 9:** Adjusted *p*–values Bergmann–Hommel procedure for scenario 8 (ref. C13).

Rank	Case	P Bergmann–Hommel
1	C3	2.45 × $10^{-08}$
2	C21	3.34 × $10^{-08}$
3	C20	1.58 × $10^{-07}$
4	C12	1.41 × $10^{-06}$
5	C2	4.05 × $10^{-06}$
6	C1	1.18 × $10^{-05}$
7	C11	6.89 × $10^{-04}$
8	C15	3.12 × $10^{-03}$
9	C10	3.62 × $10^{-03}$
10	C24	1.05 × $10^{-02}$
11	C5	3.13 × $10^{-02}$
11	C19	3.13 × $10^{-02}$
11	C27	3.13 × $10^{-02}$
12	C14	7.46 × $10^{-02}$
13	C6	7.75 × $10^{-02}$
14	C23	1.13 × $10^{-01}$
15	C25	1.49 × $10^{-01}$
16	C9	4.29 × $10^{-01}$
17	C8	5.53 × $10^{-01}$
17	C22	5.53 × $10^{-01}$
18	C4	6.15 × $10^{-01}$
19	C7	7.93 × $10^{-01}$
19	C16	7.93 × $10^{-01}$
19	C17	7.93 × $10^{-01}$
19	C18	7.93 × $10^{-01}$
19	C26	7.93 × $10^{-01}$

**Table 10:** Adjusted *p*–values Bonferroni procedure for α=0.05/27 (scenario 8)—best case C13.

Rank	Case	P Bonferroni
1	C3	3.440610 × $10^{-07}$
2	C21	4.698387 × $10^{-07}$
3	C20	2.317553 × $10^{-06}$
4	C12	2.163121 × $10^{-05}$
5	C2	6.472240 × $10^{-05}$
6	C1	1.987919 × $10^{-04}$
7	C11	1.210591 × $10^{-02}$
8	C15	6.087186 × $10^{-02}$
9	C10	7.063474 × $10^{-02}$
10	C24	2.317351 × $10^{-01}$
11	C5	7.865499 × $10^{-01}$
11	C19	7.865499 × $10^{-01}$
11	C27	7.865499 × $10^{-01}$
14	C4	1.000000 × $10^{-00}$
14	C6	1.000000 × $10^{-00}$
14	C7	1.000000 × $10^{-00}$
14	C8	1.000000 × $10^{-00}$
14	C9	1.000000 × $10^{-00}$
14	C14	1.000000 × $10^{-00}$
14	C16	1.000000 × $10^{-00}$
14	C17	1.000000 × $10^{-00}$
14	C18	1.000000 × $10^{-00}$
14	C22	1.000000 × $10^{-00}$
14	C23	1.000000 × $10^{-00}$
14	C25	1.000000 × $10^{-00}$
14	C26	1.000000 × $10^{-00}$

**Table 11:** Optimized glider headings for scenario 8.

Headings	1	2	3	4	5	6	7	8	9	10
**Indiv-1**	101.843	3.475	−2.828	3.576	1.797	−7.925	4.228	5.304	−7.232	2.118
**Indiv-2**	115.325	2.387	3.369	2.691	−14.945	−8.553	−8.460	12.263	−1.276	1.708
**Indiv-3**	−105.884	129.065	19.088	−9.868	−6.821	55.787	−11.275	17.250	8.822	−7.443

## Abbreviations

The following abbreviations are used in this manuscript:

CP	Crossover Probability
EA	Evolutionary Algorithms
FES	Fitness Evaluations
GA	Genetic Algorithm
GPP	Glider Path Planning
HF	High-Frequency
MO	Multi-Objective
MOEA	Multi-Objective Evolutionary Algorithms
MOPP	Multi-Objective Path Planning
MOOP	Multi-Objective Optimization Problems
MP	Mutation Probability
NG	Number of Generations
NSGA-II	Non-Dominated Sorting Genetic Algorithm
PS	Population Size
